# Heterogeneity of Inter-Rater Reliabilities of Grant Peer Reviews and Its Determinants: A General Estimating Equations Approach

**DOI:** 10.1371/journal.pone.0048509

**Published:** 2012-10-31

**Authors:** Rüdiger Mutz, Lutz Bornmann, Hans-Dieter Daniel

**Affiliations:** 1 Professorship for Social Psychology and Research on Higher Education, ETH Zurich, Zurich, Switzerland; 2 Division for Science and Innovation Studies, Administrative Headquarters of the Max Planck Society, Munich, Germany; 3 Evaluation Office, University of Zurich, Zurich, Switzerland; Sapienza University of Rome, Italy

## Abstract

**Background:**

One of the most important weaknesses of the peer review process is that different reviewers’ ratings of the same grant proposal typically differ. Studies on the inter-rater reliability of peer reviews mostly report only average values across all submitted proposals. But inter-rater reliabilities can vary depending on the scientific discipline or the requested grant sum, for instance.

**Goal:**

Taking the Austrian Science Fund (FWF) as an example, we aimed to investigate empirically the heterogeneity of inter-rater reliabilities (intraclass correlation) and its determinants.

**Methods:**

The data consisted of N = 8,329 proposals with N = 23,414 overall ratings by reviewers, which were statistically analyzed using the generalized estimating equations approach (GEE).

**Results:**

We found an overall intraclass correlation (ICC) of reviewer? ratings of ρ = .259 with a 95% confidence interval of [.249,.279]. In humanities the ICCs were statistically significantly higher than in all other research areas except technical sciences. The ICC in biosciences deviated statistically significantly from the average ICC. Other factors (besides the research areas), such as the grant sum requested, had negligible influence on the ICC.

**Conclusions:**

Especially in biosciences, the number of reviewers of each proposal should be increased so as to increase the ICC.

## Introduction

The legitimacy of the approval procedure at funding agencies for basic research depends strongly on whether the reliability, validity, and fairness of the procedure are guaranteed [1]. Quality control undertaken by peers in the traditional peer review of proposals for grants is essential in most research funding organizations to establish valid and evidence-based approval decisions by the board of trustees [2]. According to Marsh, Jayasinghe, and Bond [3], one of the most important weaknesses of the peer review process is that the ratings given to the same proposal by different reviewers typically differ. This results in a lack of inter-rater reliability (IRR). Cicchetti [4] defines IRR as “the extent to which two or more independent reviews of the same scientific document agree” (p. 120). Overviews of the literature on the reliability of peer reviews for grant applications[3]–[5] come to similar conclusions as those for journal peer review (e.g., [6]–[7]): There is on the average a low level of IRR.

To calculate IRR in the case of continuous ratings, intraclass correlations (ICCs) are often used; roughly speaking, the ICC is defined as a ratio of the variance of the mean ratings across all reviewers of a grant proposal and the total variance across all reviewer? ratings of a proposal.

Studies on the IRR of peer reviews mostly report average values across all submitted proposals [4]. But this can lead to biased estimation of the actual IRR, if reviewers’ ratings are not homogeneous. Reviewers in some scientific disciplines can rate proposals on average more strictly than reviewers in other scientific disciplines do (*heterogeneity with respect to the mean*). According to Marsh et al. [3], ratings of reviewers are affected by a number of covariates called bias factors – including applicant’s gender, reviewer’s gender, grant sum requested – that have nothing to do with the quality of a proposal [1]. As a result, the variance of the mean ratings as well as the total variance can also be explained by these covariates.

Over and above that, properties of grant proposals can also affect the total variance of reviewers’ ratings (*heterogeneity with respect to the variance*). For instance, it can be supposed that in the humanities and social sciences reviewers’ ratings vary more greatly than in the natural sciences. This may be due to the lack of uniform evaluation standards [8] or to greatly varying quality of proposals. Mallard et al. [9] pointed out, “noting that evaluators focus on the intellectual merits of proposals or articles provides little leverage for analyzing procedural fairness when conflicting criteria are used to define intellectual merit, as is generally the case in the social sciences and humanities” (p. 577). A greater variance of ratings in certain scientific disciplines can, but must not necessarily, be accompanied by a lower ICC. If it is found that the IRRs in the humanities and social sciences are comparable to the IRRs in the natural sciences, as Jayasinghe et al. [5] found. The higher variability of reviewers’ ratings in the humanities and social sciences is due to the more greatly varying quality of the proposals in these disciplines. Including further covariates (such as the grant sum requested, time point of the final approval decision) makes it possible to determine the specific combination of conditions that leads to differences in the variability of reviewer’s ratings. In addition to the heterogeneity of ratings with regard to the mean and variance proposals can also differ with regard to the ICC itself (*heterogeneity with respect to the ICC*). Thus, the ICCs can vary with regard to various covariates, as Marsh et al. [3] showed.

As this overview of studies on reliability shows, when examining the IRR of peer reviews using ICCs, it is necessary to include all three components (mean, variance, ICC) in the statistical analysis to obtain reliable information about the IRR.

In this study, we will determine the ICCs controlling for the specific bias factors. This means that the ICCs are calculated on condition that all proposals have the same values of the included covariates.

The generalized estimating equations approach (GEE) (especially the further [10]–[12] development of the approach by Yan and Fine [13]) makes it possible to model the heterogeneity of ICC statistically with a set of covariates while simultaneously considering the heterogeneity of variances and impacts of bias factors. However, empirical analysis of the heterogeneity of reviewers’ ratings requires a large database. We decided to conduct an empirical study of the heterogeneity of ICCs and its multiple determinants, taking as an example the Austrian Science Fund (FWF). The data consisted of all proposals of the FWF, generated by the FWF review procedure between the years 1999 and 2009; all scientific disciplines were represented in the database. This is an ideal database for the purpose of this study.

In the following, the FWF will be described in more detail and the research questions presented. The data on which the analysis was based will be characterized and the statistical approach explained. The results are then reported and discussed.

### The Austrian Science Fund (FWF)

The FWF is Austria’s central funding organization for basic research. The body responsible for funding decisions at the FWF is the board of trustees, made up of 26 elected reporters and 26 alternates [14]. For each grant application, the FWF obtains at least two international expert reviews. The number of reviewers depends on the amount of funding requested. The expert review consists (among other things) of an extensive written comment and a rating providing an overall numerical assessment of the application. At the FWF board’s decision meetings, the reporters present the written reviews and ratings of each grant application. The FWF does not enforce any quotas or specific budgets for individual scientific disciplines, and as a result, all applications from all fields and disciplines compete with one another at the five decision meetings held each year. In the period under study here (from 1999 to 2009), the approval rate of proposals was 44.2% [14].

From 1999 to 2004, the approval rate dropped continuously from 53.4% in 1999 to 36.2% in 2004; after that, it increased slightly to 42.9% in 2008 (but dropped again to 32.2% in 2009) [14]. The year 2004 thus represents a turning point in the development of the approval rate over time. One reason for this development is that in the years from 2002 to 2004, the number of grant applications and the grant sum requested exceeded the funding budget, so that the approval rate dropped from 49.2% to 36.2%. With a low approval rate, to be approved for a grant a proposal had to achieve a higher mean reviewers’ rating.

### Research Questions

Specifically, our paper addresses the following four research questions (in parentheses: in terms of GEE):

1. How reliable are the reviewers’ single ratings of the quality of the projects (that is, the overall evaluation of the proposed research by a single reviewer)?
2. Is the ICC homogeneous across all proposals, or does it vary with certain characteristics of proposals or reviewers (*intraclass correlation*)?
3. Is the total variability of reviewers’ ratings equal for all proposals, or does it vary with certain characteristics of proposals or reviewers (*variance*)? Do the ICC changes if variance heterogeneity is considered?
4. Is there any impact of covariates on reviewer? overall ratings of a proposal (*mean*)? Do the ICC changes if this impact is permitted?

## Methods

### Data and Variables

The data for this study, generated by the usual review procedure at the FWF, consisted of all proposals (N = 8,358) for individual research projects called “Stand-Alone Projects” [14] across all fields of research (6 research areas) from 1999 to 2009, which contributed to 60% of all FWF grants (“Stand-Alone Projects,” “Special Research Programs,” “Awards and Prizes,” “Transnational Funding Activities”). External reviewers (N = 18,357) (about 2 to 3 reviewers for each proposal on average) rated the proposals on a scale from 0 to 100 (from poor to excellent) in 23,977 reviews [14]. Due to missing values in the variables included in the data analysis, the effective sample (case-wise deletion) consisted of 8,329 proposals with 23,414 reviews. Given that the proportion of missing values was rather low (below 5%), we did not use any missing value treatment (e.g., missing value imputation) to complete the data [15].

The single overall rating of a proposal by each external reviewer (overall rating) provided for the outcome variable. We included in the data analyses several covariates as predictors, both on the level of proposals and on the level of reviews (see Table 1). On the level of proposals, “applicant’s gender,” “applicant’s age,” “time point of the final approval decision” by the FWF board of trustees (before 2004 or after), “requested grant sum,” and the proposal’s “research areas” were considered. Each applicant must assign her or his proposal to up to four different disciplines chosen from a list of 22 disciplines, and assign a percentage to each such that the percentages sum to 100%. The scientific discipline with the highest proportion is defined by the FWF as the proposal’s primary discipline, which is again classified by the FWF into one of six research areas (Table 1). On the level of reviewers, the “reviewer’s gender” and the “continent of the reviewer’s address” were included. Altogether, the selected variables were factors that are typically examined in peer review studies [1].

**Table 1:** Summary description of the data from the Austrian Science Fund (N_p_ = 8,329 grant proposals, N_r_ = 23,414 reviews).

Variables	Coding	N	%	Mean	SD	MIN	MAX
*Proposal attributes*							
Research areas							
Biosciences	1/0	1628	19.6				
Humanities	1/0	1413	17.0				
Human medicine	1/0	1621	19.5				
Natural sciences	1/0	2450	29.4				
Social sciences	1/0	697	8.4				
Technical sciences	−1/0	520	6.2				
Applicant’s gender							
Female	1	1473	17.7				
Male	−1	6856	82.3				
Applicant’s age				46.7	9.8	23	87
Time point of the final approval decision							
2004 and after	1	4,994	60.0				
Before the year 2004	−1	3,335	40.0				
Requested grant sum (100,000 euros)				2.45	1.16	0.04	8.13
*Review attributes*							
Overall rating				81.8	15.6	0	100
Reviewer’s gender							
Female	1	3,068	13.2				
Male	−1	20,246	86.8				
Continent of the reviewer’s address							
Europe	1/0	14,291	60.7				
North America	1/0	7,575	32.2				
Other	−1/0	1,664	7.1				

Note. Effect coding is used for the categorical variables. For categories coded with −1, no parameter could be estimated, but a parameter β_c_ could be numerically obtained from the other parameters (β_c_ = −Σβ_j = 1…(c−1)_). SD  =  the standard deviation, MIN and MAX stand for minimum and maximum.

The categorical covariates (e.g., “applicant’s gender,” “research areas”) were effect coded to interpret the parameters in the regression part of the model as a deviation from the grand mean, which is estimated by the intercept.

### Statistical Analysis

The unconditional ICC (ρ) as a measure of single IRR, or intra-proposal correlation, was derived from estimation of variance components using two-level models [5], [16]–[20], especially a random-intercept model, with reviews as level-1 and, proposals as level-2 units (reviews are nested within proposals). The ICC is given by.
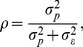
(1)where σ^2^_p_ is the between-proposal variance and σ^2^_ε_ is the within-proposal variance or residual variance. An ICC of.30 means that on the average across the whole data, two reviews of the same proposal are correlated with ρ = .30. Further possible measurement dependencies due to the fact that the same reviewer assesses several proposals can be neglected, given the small proportions of reviewers that assessed more than one proposal in the period under study.

In addition, standard errors and corresponding confidence intervals for the parameters are calculated. For the ICC, different procedures to calculate standard errors or confidence intervals are discussed (e.g., [20]), such as classical F-test procedures [19], bootstrap procedures [21], latent variable modeling [22], and profile-likelihood based methods [23]. We decided to use latent variable modeling and F-test based procedures; they are well founded and very common, and the confidence intervals can be calculated easily using common statistical software packages like SAS [24].

The ICC was entered into Eq. (1) as a measure of single IRR. For research funding organizations and their decision making, it is more important to know how reliable the mean ratings are across all reviewers of a proposal. Final approval decisions of the organizations (including approval decisions by the FWF board of trustees) are strongly based on the mean ratings of a proposal. By using the Spearman-Brown prophecy formula from classical test theory ([19], p. 426) the ρ_M_, or ICC(1, k) in the notation by Shrout and Fleiss [19], can easily be derived from the ICC (ρ) as follows:
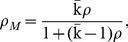
(2)where 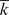 is the average number of reviews of a proposal: 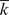 = 1/(N−1)(Σk−Σk^2^/Σk) [26], where k is the number of reviewers for a single proposal and N is the total number of proposals. For instance, for a single IRR ρ = .30 and 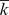 = 3 reviewers for each proposal, the IRR of the mean ratings amounts to ρ_M_ = .56, which is quite a bit higher than the single IRR. To calculate ρ_M_, we used the average number of reviews of a grant proposal at FWF. On average, a proposal submitted to the FWF is reviewed by 2.82 reviewers.

The ICC might vary according to different combinations of characteristics of reviews or proposals. To model heterogeneous ICCs *second-order generalized estimating equations* have been discussed as a further development of the well-known generalized estimating equations (GEE) approach [10]–[12], [25]. Whereas multilevel models estimate both the random part and – conditioned on the random part – the fixed-effects or mean part of the model, the GEE approach focuses mainly on the mean part of the multilevel model (marginal model, population-average model). The random part and its variance components are considered to be a nuisance characteristic, to accurately estimate the mean model and its standard errors.

The GEE approach derives from the generalized linear model, which incorporates all types of dependent variables (count, binary, ordinal …) using certain link functions. By the way, the mean model of GEE with a link function other than identity (i.e., continuous normally distributed variable) might deviate from the fixed-effects part of an ordinary multilevel model. Simple assumptions of the dependency of measurements (i.e., intra-proposal correlation) represented by a “working correlation matrix” make parameter estimations much faster than classical multilevel models might do [18]. The second-order GEE approach introduced by Yan and Fine [13] extends the classical GEE approach [26]–[28]. Additional to the link function of the mean model, separate link functions for the variance and for the ICC were introduced to connect mean, variance, and ICC with different sets of covariates as predictors [13].

With respect to our data, the proposals provide for the clusters, and the reviewer? ratings of a proposal provide for the level-1 units nested within the proposals. Due to the huge number of different reviewers it is not sensible to take into account the reviewer as an additional factor or level in multilevel modeling.

Let Y_ij_ be the single rating j, j = 1,…n_i_, from proposal i = 1, … K, the vector **µ**_i_ (n_i_×1) be the expectation of Y_ij_ conditioning on the matrix of p predictors **X**_1i_ (n_i_×p). Additionally, the vector φ_i_ (n_i_×1) denotes the vector of variances conditioning on the matrix of r predictors **X**_2i_ (n_i_×r), and **ρ**_i_ (n_i_ (n_i_−1)/2) denotes the vector of intraclass correlations conditioning on the matrix of q predictors **X**_3i_ (n_i_ (n_i_−1)/2×q). According to this definition, the following link functions can be defined:
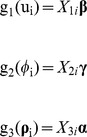
(3)


The identity function (g_1_) was chosen as link function for the fixed-effects or mean model (continuous normally distributed variable), and for the variance the log-function (g_2_). For the ICC we used a modified Fisher-z transformation (g_3_), which guarantees that the ICC vary within the interval [−1, 1]. With identity as link function for the mean, the vector φ_i_ denotes variances. In all other nonlinear or categorical cases vector φ_i_ denotes a vector of scale parameters (i.e., overdispersion). The inverse link function g_3_^−1^ for the ICC between the overall ratings of two reviewers r and s within a proposal i is ([13], p. 862)
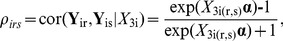
(4)where **X**_3i(r,s)_ is the row in **X**_3i_, which corresponds to the ICC of the overall ratings of reviewers **Y**_is_ and **Y**_ir_; “exp” denotes the exponential function e^x^. A convenient set of estimating equations can be defined to estimate the model ([13], p. 863). Crespi, Wong, and Mishra [26] showed that inferences in cluster-randomized trials can be improved, if heterogeneous ICCs are accurately modeled.

We used Wald tests (β/standard error for H_0_: β = 0) to test the statistical significance of the parameters. To avoid common problems of statistical testing [29], we report here the recalculated “effect sizes” (e.g., ICC) beside the parameters and standard errors. In statistical testing it is usual to discuss the statistical power of a test, that is the probability to reject the null hypothesis, if it is actually false in the population. Due to the huge sample size, the statistical power of the test might be high. Therefore, even very low effects might be detected, if there are some.

### Modeling Strategy

The data analysis was conducted in two steps: First, for a descriptive analysis, the ICC and the corresponding confidence interval were calculated for both the overall data set and also for the separate research areas and the separate years of the final decision by the FWF board of trustees. For comparing the ICCs across the research areas and years of the final decision, we used Goldstein-adjusted confidence intervals, which were originally developed to compare means [30]–[31]. This adjustment allows interpretation of non-overlapping intervals as statistically significant differences (α = .05) between the research areas or years of the final decision in the level of ICC.

In the second step, four second-order GEE models were estimated to determine the influence of various restrictions on the size of the ICC, whereby restrictive assumptions of the previous models were successively suspended.

A most restricted base model (Model 0) was fitted that assumes homogeneity with respect to all three components (mean, variance, ICC), with three intercepts for each component. However, if there is a misspecification of the ICC component, only the mean and variance parameters are robust and consistent estimators. Thus, the ICC should be interpreted with caution [13].

A second model (model 1) was estimated that takes into account the heterogeneity of the ICC with a regression model for correlations (Eq. (3), g_3_(**α**_i_)), whereas all other components as mean and variance were assumed to be constant or homogeneous across all proposals (Eq. (3), g_1_(µ_i_)).

The strong restriction of variance homogeneity across proposals was suspended in favor of a further model (model 2) that allows, in addition to model 1, the variances differing according to the set of covariates mentioned above with possible impacts on the size of ICC (Eq. (3), g_2_(**γ**_i_)). With respect to the definition of ICC in Eq. (1), if the denominator (total variance) increases and the between-proposal variance is held constant, the ICC decreases, and vice versa – if the denominator decreases, the ICC increases.

In the last step, a GEE (model 3) was estimated that not only allows variance heterogeneity but also allows that the single ratings are adjusted for a set of covariates, quite comparable to an ordinary regression model (Eq. (3), g_1_(µ_i_)). Therefore, the estimated ICCs are nothing but the ICC of the residuals, which are adjusted for mean differences of a set of covariates (bias factors). For instance, if scientific disciplines differ in the mean ratings, for example because of leniency effects, the ICCs are adjusted for this impact by including the scientific disciplines as covariates in the mean component of the GEE.

We assume that the results of the first analysis step (descriptive analysis, see above) do not have to agree completely with the results of the second step for two reasons: For one, in the first step the ICCs were calculated for different groups, or parts, of the data (e.g., research areas); in the second step, however, in the context of GEE the ICCs were estimated for the whole data with varying restrictive assumptions. For another, in GEE several covariates were used in a multiple regression model for mean, variance, and ICC, whereas the calculation of ICC in the first analysis step considered only one factor.

### Software

The ICCs were calculated using the SAS procedure “proc mixed” [24]. A self-programmed SAS macro was used to estimate the F-test based confidence intervals according to Shrout and Fleiss [19]. The second-order GEE was calculated using the R package “geepack” [13], [28], [32].

## Results

### Inter-rater Reliability

The ICC (single IRR) for the whole data set of 23,414 reviewers is ρ = .259 with an F-test based 95%-confidence interval of [.249,.279] and a latent-variable based confidence interval of [.243,.275]. Thus, the overall ratings of the same proposal correlate about.26 on the average across all reviewers, or 26% of the total variance of overall ratings is explained by proposals. Whereas the simple ICC ρ says something about single ratings, the reliability of the mean ratings averaged across all reviewers of a proposal ρ_m_ says more about the reliability of the FWF peer review procedure (see above). The ICC of mean ratings amounts to ρ_m_ = .495 (with 2.82 reviews per proposal) and is quite higher than the single IRR.

**Figure 1:**
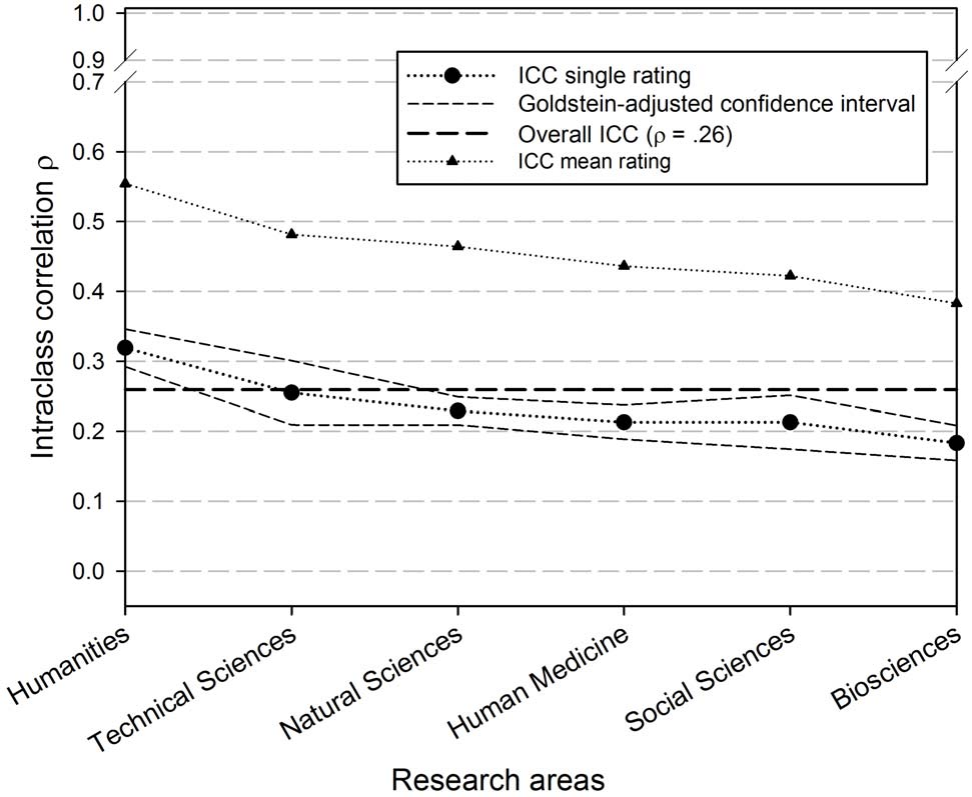
Intraclass correlations, overall and for the separate research areas. Lines are shown as dotted because research area is categorical, so interpolation between research areas is not intended.

Figure 1 shows the ICC for the whole population, the different single ICCs with confidence intervals, and the ICCs of the mean ratings. The ICCs or single-rater reliabilities vary strongly between the research areas from ρ = .183 (biosciences) to ρ = .319 (humanities). Due to non-overlapping confidence intervals, the ICC of humanities (ρ = .319) differs statistically significantly from natural sciences (ρ = .255), human medicine (ρ = .229), social sciences (ρ = .213), and biosciences (ρ = .183). The ICC of the mean ratings is quite a bit higher and varies between.383 (biosciences) and.55 (humanities).

**Figure 2:**
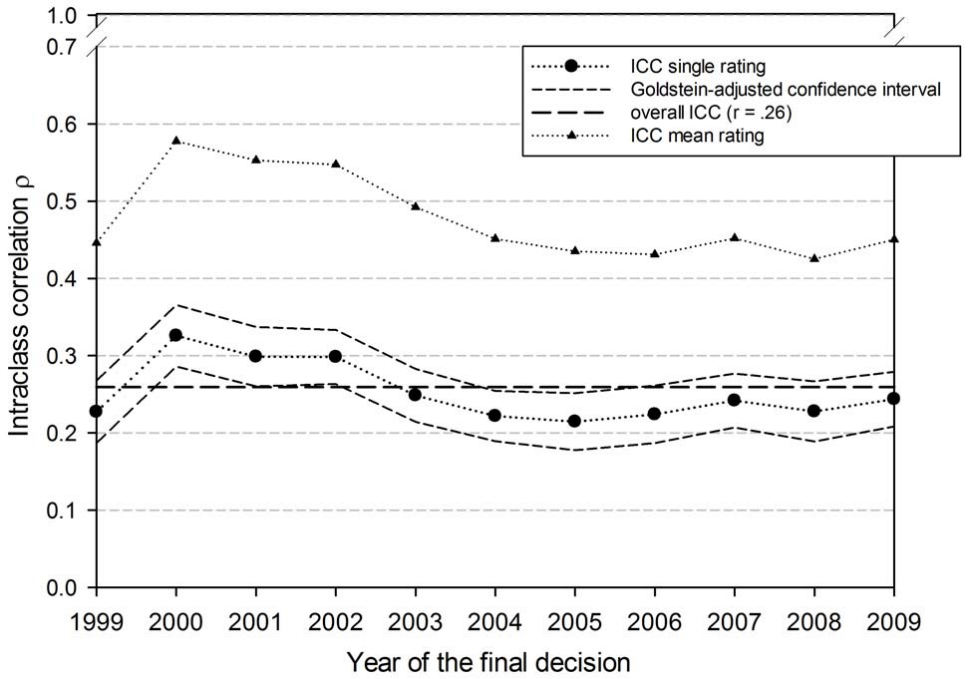
Intraclass correlations, overall and for the separate years of the final decision by the board of trustees of the Austrian Science Fund.

Figure 2 shows the same coefficients broken down by year of the final decision. In contrast to the field-specific ICCs, the ICCs do not vary strongly across the years. There are statistically significant differences (non-overlapping intervals) only between the year 2000 (ρ = .326) and the years 2004 and 2005 (ρ = .215, ρ = .224).

### Determinants of ICC, Variance, and Mean

Due to its huge size the results of the GEE analysis are presented in three tables (Table 2, 3, and 4), one table for each component (ICC, variance, mean). In model 1 only the regression of the ICC (i.e. single IRR) on its determinants is considered (Table 2). This same regression is considered, either if the model is controlled for the determinants of the variance (model 2, Table 3), or if it is controlled for both the variance and the mean model (model 3, Table 4). Statistically significant predictors in the models indicate heterogeneity of the parameters (ICC, variance). The parameters (intercept and slope) are transformed into ICCs using the inverse link function (Eq. (4)) or into variances using the exponential function (e^x^). Unfortunately, up to now there is lack of criteria ([12], p. 62f) such as information criteria (like Akaike’s information criterion) to compare different models, and, therefore, no such criteria have been implemented as yet in the R procedure “geepack.”

**Table 2:** Results I of fitting generalized estimating equations (GEE) models (intraclass correlation parameters) to the data from the Austrian Science Fund, with standard errors in brackets.

Predictors	Par	Model 0		Model 1		Model 2		Model 3	
		No predictors		Only predictors for ICC		Predictors for ICC and variance		Predictors for ICC, variance, and mean	
ICC		Estim	ρ	Estim	ρ	Estim	ρ	Estim	ρ
Intercept	α_0_	0.45* (0.02)	.22	0.49* (0.03)	.24	0.45* (0.03)	.22	0.40* (0.03)	.20
Biosciences	α_1_			−0.22* (0.04)	.13	−0.15* (0.04)	.15	−0.13* (0.03)	.14
Humanities	α_2_			0.30* (0.09)	.38	0.24* (0.06)	.34	0.20* (0.06)	.29
Human medicine	α_3_			−0.05 (0.05)	.22	−0.06 (0.04)	.19	−0.07 (0.04)	.16
Natural sciences	α_4_			−0.17* (0.04)	.16	−0.04 (0.03)	.21	−0.05 (0.03)	.17
Social sciences	α_5_			0.22* (0.10)	.34	0.04 (0.06)	.23	0.02 (0.06)	.21
Technical Sciences	(α_6_)			−0.08	.20	−0.03	.21	−0.03	.18
Time point (1 = ‘≥2004’)	α_7_			−0.02 (0.02)	.23	−0.03 (0.02)	.21	−0.01 (0.02)	.19
Request. grant sum (100,000 euros)	α_8_			0.00 (0.02)	.24	0.05* (0.02)	.25	0.03 (0.02)	.21
Applicant’s gender (1 = women)	α_9_			−0.02 (0.03)	.23	−0.02 (0.02)	.21	−0.01 (0.03)	.19
Applicant’s age/10	α_10_			0.02 (0.02)	.25	0.03 (0.02)	.24	0.00 (0.02)	.20

Note. Par  =  parameters, Estim  =  estimate (Fisher z for ICC), ρ  =  ICC for a one-unit change in the predictor variable. Parameter in brackets indicates the category coded with −1. N_p_ = 8,329 proposals, N_r_ = 23,414 reviews.

* p<.05 (Wald test).

**Table 3:** Results II of fitting generalized estimating equations (GEE) models (variance parameters) to the data from the Austrian Science Fund, with standard errors in brackets.

Predictors	Par	Model 0		Model 1		Model 2		Model 3	
		No predictors		Only predictors for ICC		Predictors for ICC and variance		Predictors for ICC, variance, and mean	
Variance		Estim	SD	Estim	SD	Estim	SD	Estim	SD
Intercept	γ_0_	5.49* (0.02)	15.6	5.49* (0.02)	15.6	5.41* (0.03)	15.0	5.37* (0.03)	14.6
Biosciences	γ_1_					−0.17* (0.03)	13.8	−0.15* (0.04)	13.6
Humanities	γ_2_					0.06 (0.04)	15.4	0.06 (0.05)	15.1
Human medicine	γ_3_					0.11* (0.03)	17.2	0.08* (0.03)	15.2
Natural sciences	γ_4_					−0.28* (0.03)	13.0	−0.26* (0.03)	12.8
Social sciences	γ_5_					0.34* (0.05)	17.7	0.31* (0.04)	17.1
Technical sciences	(γ_6_)					−0.05	14.6	−0.04	14.4
Time point (1 = ‘≥2004’)	γ_7_					0.03* (0.02)	15.2	0.04* (0.02)	14.9
Request. grant sum (100,000 euros)	γ_8_					−0.08* (0.02)	14.4	−0.08* (0.02)	14.1
Applicant’s gender (1 = women)	γ_9_					−0.00 (0.02)	15.0	−0.00 (0.02)	14.6
Applicant’s age/10	γ_11_					0.00 (0.02)	15.0	0.01 (0.02)	14.7
Reviewer’s gender (1 = women)	γ_12_					−0.01 (0.02)	14.9	−0.01 (0.02)	14.6
Reviewer’s continent									
Europe	γ_13_					0.14* (0.02)	16.0	0.15* (0.03)	15.7
North America	γ_14_					0.11* (0.02)	15.8	0.12* (0.03)	15.5
Others	(γ_15_)					−0.25	13.2	−0.26	12.9

Note. Par  =  parameters, Estim  =  estimate (log for variance), SD  =  standard deviation estimates for a one unit change in the predictor variable. Parameters in brackets indicate the category coded with −1. N_p_ = 8,329 proposals, N_r_ = 23,414 reviews.

* p<.05 (Wald test).

**Table 4:** Results III of fitting generalized estimating equations (GEE models) (mean model) to the data from the Austrian Science Fund.

Predictors	Par	Model 0		Model 1		Model 2		Model 3	
		No predictors		Only predictors for ICC		Predictors for ICC and variance		Predictors for ICC, variance, and mean	
		Estim	SE	Estim	SE	Estim	SE	Estim	SE
Intercept	β_0_	81.59*	0.13	81.60*	0.12	81.45*	0.12	81.34*	0.22
Biosciences	β_1_							0.76*	0.25
Humanities	β_2_							4.40*	0.33
Human medicine	β_3_							−2.88*	0.28
Natural sciences	β_4_							2.18*	0.22
Social sciences	β_5_							−3.39*	0.44
Technical sciences	(β_6_)							1.07	
Time point (1 = ‘≥2004’)	β_7_							0.48*	0.12
Request. grant sum (100,000 euros)	β_8_							1.04*	0.12
Applicant’s gender (1 = women)	β_9_							−0.37*	0.16
Applicant’s age/10	β_10_							0.42*	0.13
Reviewer’s gender (1 = women)	β_11_							−0.07	0.15
Reviewer’s continent	β_12_								
Europe	β_13_							−0.84*	0.15
North America	β_14_							−0.72*	0.16
Other	(β_15_)							1.57	

Note. Estim  =  estimate, SE  =  standard error. Parameters in brackets indicate the category coded with −1. N_p_ = 8,329 proposals, N_r_ = 23,414 reviews.

* p<.05 (Wald test).

The starting model (model 0, Tables 1, 2, 3) shows that the mean overall rating is 81.59 (β_0_), the standard deviation of the ratings is 15.6 (γ_0_ = 5.59), and the mean ICC is.22 (α_0_ = .45), which differ slightly from the ICC (ρ = .26), reported in Figure 1 (which should be interpreted with caution, see section “modeling strategy”). In model 1 (Table 2) the central statistically significant predictors of the ICCs are the research areas. Whereas the ICCs for biosciences and natural sciences decrease (.13,.16) in comparison to the mean ICC, the ICCs of humanities and social sciences increases (.38,.34). Technical sciences and human medicine show negligible deviations from the average ICC. Further, there is no empirical evidence for the impact of any other property of a proposal as, for instance, applican?s age or “applican?s gender” on the single ICC.

With Model 2 the restrictive assumption of variance homogeneity no longer holds. As Table 3 shows, the variance and standard deviation, respectively, vary considerably and statistically significantly with respect to “research areas” and “reviewer’s continent” and less so with respect to “time point of the final approval decision” and “requested grant sum.” Whereas the standard deviation of the overall ratings in natural sciences and biosciences (13.0, 13.8) fall considerably short of the mean standard deviation of 15.0, the standard deviation of the social sciences (17.7) considerably exceeds the mean standard deviation. Reviewers located in Europe or North America give much more heterogeneous ratings (higher variance) than reviewers located in other regions. With higher requested grant sum (above 100,000 euros) the variability of the ratings decreases (γ_8_ = −0.08).

However, suspending the restriction of equal variances results in a shrinking of the ICCs toward the mean ICC of.22. Out of the research areas only two areas show a statistically significant deviation from the average IRR (Model 2), humanities with a positive deviation, and biosciences with a negative deviation from the average IRR.

The greatest change in the ICC is found for the social sciences – from.34 (model 1) to.23 (model 2). Together with the higher than average variance, which is the denominator in the ICC formula (Eq. 1) (var = σ^2^_ε_ + σ^2^_p_), it is found that the drop of the ICC in the social sciences is due more to the higher error variance σ^2^_ε_ and less to the systematic variance σ_p_ between proposals that reflects differences in the quality of the proposals. Therefore, in the social sciences reviewers’ ratings are considerably more heterogeneous than in other research areas, especially the natural sciences, without this higher variability necessarily reflecting greater differences in the quality of the proposals. However, in absolute terms, the quality differences in the proposals in the social sciences (expressed in variance units) are almost twice as high as the quality differences in the natural sciences, for instance. The variance between proposals σ^2^_p_ is 71.2 for the social sciences and only 35.5 for the natural sciences, when we use a simple conversion of Eq. 1 to calculate σ^2^_p_ from the estimated parameters (σ^2^_p_ = ρ*(σ^2^_ε_ + σ^2^_p_) = ρ* variance). Interestingly, the variability between proposals, σ^2^_p_, is the highest in the humanities (80.6), but with a considerably lower variance (13.8^2^ = 237.1) than in the social sciences (17.7^2^ = 313.7). For this reason, the single-rater reliability in the humanities is clearly higher than that in the social sciences.

In addition, controlling for variance determinants reveals that in comparison to model 1, “requested grant sum” now has a statistically significant impact on the ICC. If the grant sum requested is 100,000 euros higher than the average grant sum, then the ICC increases slightly from.22 (intercept) to.25.

In addition to model 2 in model 3 the overall rating was regressed on a set of covariates (Eq. (3), g_1_(µ_i_)) comparable to an ordinary regression or fixed-effects model (Table 4). In sum, there are many statistically significant predictors with small effect sizes. The rating levels in humanities (natural sciences) are 4.40 (2.18) grade points higher (0–100 rating scale) than the overall mean rating, whereas the ratings in the social sciences (human medicine) fall on the average −3.39 (−2.88) short of the overall mean rating. The other statistically significant predictors have negligible effects around and below 1 grade point (“time point of the final approval decision,” “requested grant sum,” “applicant’s age”), with one exception. If the reviewer’s continent was not Europe or North America but “other,” the corresponding reviews are 1.57 grade points more favorable than the average of 81.34.

Including predictors in the mean model results in a further shrinking of both the ICC and the variances of the residuals. The amount of reduction of variance from model 2 to model 3 (Table 3) reflects the amount of variance that is explained by the predictors of the mean model. For the social sciences, for instance, the variance drops from 13.0^2^ = 313.7 to 12.8^2^ = 292.9– that is, 100*(313.7–292.9)/313.7 = 6.6% of the variance in the social sciences is explained by the mean model. Overall, 0% to 8% of the variances of the different factors are explained by the predictors of the mean model.

## Discussion

Taking the peer review process of the FWF as an example, we focused in this contribution on the IRR of reviewers’ ratings of a proposal, especially its heterogeneity with respect to certain covariates (e.g., research areas). In sum, our study yielded the following findings as answers on the research questions:

1. *How reliable are the reviewers’ single ratings of the quality of the projects (that is, the overall evaluation of the proposed research by a single reviewer)?* In agreement with other peer review studies, we found for the FWF a low overall IRR of reviewers’ ratings of ρ = .259 (single-rater) with a F-test based confidence interval of [.249,.279]. Thus, the overall ratings of two reviewers of the same proposal correlate about.26 on the average across all reviewers. The ICC of the mean rating aggregated across all ratings of a proposal was ρ_m_ = .495. For external reviewer ratings of the Australian Research Council’s large grant program, Jayasinghe et al. [5] found single IRRs of.21 for the social sciences and humanities and.19 for science.
2. *Is the ICC homogeneous across all proposals, or does it vary with certain characteristics of proposals or reviewers?* Across humanities the ICC (IRR of single rating and of mean rating) was actually higher than in all other research areas. The ICC of biosciences deviated statistically significantly negatively from the average ICC across all disciplines. Other factors, including “time point of the final decision,” “applicant’s age,” or “applicant’s gender” can be neglected.
3. *Is the total variability of reviewers’ ratings equal for all proposals, or does it vary with certain characteristics of proposals or reviewers? Do the ICC changes if variance heterogeneity is considered?* The total variance varied considerably and statistically significantly with respect to the research areas and to a minor extent with respect to “time point of the final decision,” “requested grant sum,” and “continent of reviewer’s address.” Suspending the restriction of equal variances resulted in ICCs shrinking to the mean ICC, especially the ICCs in the social sciences (from.34 to.23). In the social sciences (standard deviation = 17.7) the reviewers’ ratings were considerably more heterogeneous than in the other research areas, especially in the natural sciences (13.2), without this higher variability necessarily reflecting greater differences in the quality of the proposals (σ^2^_p_). However, in absolute terms, the quality differences in the proposals in the social sciences were almost twice as high as the quality differences in the natural sciences. More divergent evaluation standards in the social sciences than in the natural sciences might turn into higher variability of the overall ratings of a proposal [8].
4. *Is there any impact of covariates on reviewers’ overall ratings of a proposal? Do the ICC changes if this impact is permitted?* We found statistically significant influences of several predictors on the reviewers’ overall ratings, but the associated effect sizes were small, except for the research areas. When predictors were included in the mean model, the ICCs of the residuals became smaller. Further, the groups of disciplines differed in the rating level. The rating levels in the humanities (natural sciences) were 4.40 (2.18) grade points higher (0–100 rating scale) than the overall mean rating, whereas the ratings of social sciences (human medicine) fell on the average −3.39 (−2.88) short of the overall mean rating.

Overall, our analyses show that the ICCs vary very greatly for the FWF peer review procedure; therefore, average values for the whole population of all grant applications do not provide a very differentiated picture of the reliability. As this study showed, the second-order GEE approach [13] used here is very promising for peer review research due to its property of not only modeling the ICCs but also concurrently modeling the variability and the mean of the outcome. The approach makes it possible to reveal differences between research areas in ICCs and also model differences in variances. Crespi, Wong, and Mishra [26] also showed that inferences in cluster-randomized trials can be improved, if heterogeneous ICCs are accurately modeled.

However, GEE has also its limitations: (1) GEE was developed mainly to estimate a population-average model for categorical variables in the case of dependency of measurements based on a simple ICC matrix or “working correlation matrix” in terms of GEE, (2) There is a lack of simulation studies showing how the model behaves under certain sampling conditions (e.g., different sample sizes, amount of heterogeneity of variances), and (3) Criteria for model comparison or goodness-of-fit indices, such as the QIC, which was developed for the ordinary GEE approach, are still lacking [12], [33]–[35].

Based on this study, the following recommendations can be made with regard to the peer review procedure at the FWF:

1. For some research areas (e.g., biosciences) with a comparatively low single ICC of ρ = .183, consider increasing the number of reviewers to three or four, since the results of this study showed that already with three reviewers, the IRR of the mean rating is quite higher (see Figure 1).
2. Since reviewers in the social sciences make very heterogeneous ratings, it should be examined whether the variability of the overall ratings could be reduced by making the rating criteria more precise or by modifying the selection of reviewers (e.g., applicant and reviewers share the same research paradigm), which would probably result in higher IRR.
3. As the reviewers in the different research areas give very different ratings to the grant applications, discipline-specific thresholds for mean ratings should be introduced for approving grant applications.
